# Study protocol for ELders AT Ease (ELATE): a cluster randomised controlled trial of cognitive behaviour therapy to reduce depressive symptoms in aged care residents

**DOI:** 10.1186/s12877-023-04257-7

**Published:** 2023-09-12

**Authors:** Sunil Bhar, Tanya E. Davison, Penelope Schofield, Stephen Quinn, Julie Ratcliffe, Joanna M. Waloszek, Sofie Dunkerley, Mark Silver, Jennifer Linossier, Deborah Koder, Rebecca Collins, Rachel Milte

**Affiliations:** 1 Department of Psychological Sciences, School of Health Sciences, Swinburne University of Technology, PO Box 218, H99, Hawthorn, VIC 3122 Australia; 2 Silverchain, Osborne Park, WA Australia; 3 Iverson Health Innovation Research Institute, Swinburne University of Technology, Hawthorn, VIC Australia; 4 Health Services Research and Implementation Sciences, Peter MacCallum Cancer Centre, Melbourne, VIC Australia; 5 Sir Peter MacCallum Department of Oncology, The University of Melbourne, Parkville, VIC Australia; 6 Department of Health Sciences and Biostatistics, School of Health Sciences, Swinburne University of Technology, Hawthorn, VIC Australia; 7 Caring Futures Institute, Flinders University, Adelaide, SA Australia

**Keywords:** Depression, Anxiety, Suicide ideation, Cognitive behavior therapy, Nursing home, Residential aged care facility

## Abstract

**Background:**

This protocol describes a study of the effectiveness of cognitive behaviour therapy (CBT) for reducing depressive symptoms in older adults living in residential aged care (RAC) facilities in Australia. Depressive symptoms are highly prevalent in this population, yet the benefits of CBT for reducing such symptoms in RAC facilities have not been widely investigated. Elders at Ease (ELATE) is a 16-session CBT intervention designed for implementation in RAC facilities. The intervention includes cognitive, behavioural and reminiscence strategies and is delivered by mental health trainees (MHTs) in collaboration with RAC facility staff and residents’ family.

**Methods and analysis:**

ELATE will be evaluated using a cluster randomised trial comparing outcomes for residents who participate in the intervention with those living in usual care control facilities. The participants are RAC residents aged 65 years or above, with depressive symptoms (Patient Health Questionnaire-2 ≥ 3) and normal cognition or mild cognitive impairment (Standardised Mini Mental Status Examination ≥ 21). They are assessed at four time points: baseline prior to randomisation (T1), mid-treatment (T2; 2.5 months post randomisation), post-treatment (T3; 5 months post-randomisation) and 3-month follow-up (T4; 8 months post randomisation). The primary outcome is change in depressive symptoms between T1 and T3. Secondary outcomes are depressive symptoms at T4, anxiety, suicide ideation, sleep problems, quality of life, staff and family knowledge of late-life depression, stress levels and efficacy in caring for residents, and MHT levels of geropsychology competencies. Residents receiving the intervention are hypothesised to report *a greater decrease in depressive symptoms* between T1 and T3 compared to residents receiving usual care. The primary analysis is a regression, clustered over site to account for correlated readings, and independent variables are condition and depressive symptoms at T1. A cost-utility analysis is also undertaken.

**Discussion:**

ELATE is a comprehensive CBT intervention for reducing depressive symptoms in RAC residents. It is designed to be implemented in collaboration with facility staff and residents’ families, individually tailored to residents with normal cognition to mild cognitive impairment and delivered by trainee therapists. ELATE offers a model that may be widely applicable across the RAC sector.

**Trial registration:**

Trial registered with the Australian and New Zealand Clinical Trial Registry (ANZCTR) Number ACTRN12619001037190, prospectively registered on 22 July 2019.

**Supplementary Information:**

The online version contains supplementary material available at 10.1186/s12877-023-04257-7.

## Background

The prevalence of depressive symptoms is high in residential aged care (RAC) facilities. Australian records indicate that up to 52% of RAC residents have clinically significant depressive symptoms [1, 2], a substantially higher rate compared to that in the general community of older adults [3]. Similarly, in the United Kingdom, the prevalence of such symptoms among RAC residents is twice as high as that among community-dwelling older adults [4]. Depressive symptoms among residents is associated with anxiety [5], suicide ideation [6], poor sleep [7] and poor quality of life [8, 9], highlighting the importance of developing effective treatments for such symptoms.

Current treatment approaches for late-life depressive symptoms include pharmacological and non-pharmacological interventions [10]. Antidepressant medication remains the most common intervention in RAC settings [11, 12], despite concerns raised by experts about over-prescription practices, poor efficacy and side effects of these medications for residents [13]. The over-reliance on antidepressants is reflective of the limited access that residents have to psychological services. Psychologists and social workers are rarely employed in Australian RAC facilities [14], and aged care staff are poorly trained to detect or manage depressive symptoms of residents [15, 16]. Less than 3% of RAC residents in Australia receive government subsidised mental health services [17], suggesting that such services are poorly accessible to such populations.

Psychological strategies including behavioural strategies, cognitive techniques and reminiscence are effective approaches for reducing late-life depressive symptoms [18, 19], and so are viable alternatives or adjuncts to antidepressants. Behavioural strategies include activity scheduling, relaxation, problem solving and behaviour modification strategies [20]. Cognitive techniques assist individuals to identify and modify maladaptive patterns of thinking [21, 22]. Reminiscence involves recounting and sharing memories of enjoyable experiences, important life events, and problem solving success [23]. Despite their effectiveness, they have not been widely evaluated for RAC populations. Research on such strategies has mostly drawn on community samples; for example, in a recent review of 68 randomised control trials of psychological interventions from 1982–2022 addressing late-life depressive symptoms, only 3 were conducted in RAC settings [18].

Generalising findings from community to RAC settings is problematic as residents are older, more cognitively and physically impaired and more dependent, compared to community dwelling older adults [24, 25]. Although there is preliminary evidence that psychological interventions are helpful for depressive symptoms in RAC, there are few high-quality trials [26–29]. Trial samples are typically small, and there are few validated interventions for RAC populations. Further, many existing psychological interventions rely on skilled mental health clinicians, and so are not feasible for widespread use in RAC facilities given the critical shortage of such specialists in the sector, and poor capacity among facility staff to detect and manage mental health symptoms among residents [15, 30].

We developed a psychological intervention model called Elders at Ease (ELATE), specifically designed for delivery in RAC facilities [31–33]. The intervention is especially applicable in the RAC sector for four reasons. First, it is *systemic*, in that it involves family and aged care staff in treatment delivery. Previous trials have shown that family and staff involvement in resident care is associated with positive mental health outcomes for residents [26, 34]. A number of treatment approaches have delivered treatment in collaboration with facility staff [20, 35–37], but none to date have also involved families for specifically addressing depressive symptoms among residents. Families are involved in the care of residents [38], but remain an under-utilised resource for residents undergoing psychological interventions. In ELATE, staff and family are provided training and support to detect and manage depressive symptoms in residents, consulted about treatment plans and collaborate with therapists to deliver treatment to residents.

Second, the intervention is *integrative*, as it draws on three types of evidence-based strategies – behavioural, cognitive and reminiscence – to reduce late-life depressive symptoms. The inclusion of multiple strategies is purported to make the intervention adaptable to resident capabilities and preferences. For example, behavioural and reminiscence strategies may be more feasible than cognitive strategies for residents with cognitive impairment. Reminiscence strategies may be more engaging than cognitive or behavioural strategies for residents who are reluctant to discuss problems. Cognitive strategies may be especially relevant to help residents with normal cognition to reframe perceptions and develop more helpful patterns of thinking.

Third, the intervention is designed to be *delivered by mental health trainees (MHTs)* under supervision of experienced practitioners, thus providing a possible cost-effective model of mental health care in RAC facilities. The use of trainees may also represent a creative method for making psychological treatments available to RAC residents given the critical shortage of a mental health care workforce in aged care. Most studies evaluating psychological treatments for depressive symptoms in residents have used qualified clinicians [37, 39–41]. Of the few that have used students as therapists in RAC settings [20, 22], none to our knowledge have conducted an economic evaluation of the model to examine the cost effectiveness of this model for the RAC sector.

Fourth, it addresses the *diverse needs of various stakeholders simultaneously*. It upskills facility staff, supports family members in caring for residents, and delivers evidence-based psychological interventions to residents. MHTs receive training in geropsychology, thus building capacity for the future workforce to respond to the ageing population.

### Study aims

The primary aim of this study is to examine the effectiveness of ELATE for reducing depressive symptoms in older adults living in RAC facilities. The primary hypothesis is that residents receiving the intervention will report a greater decrease in depressive symptoms between baseline and post-treatment compared to residents receiving usual care.

Secondary aims of the study are to: a) examine the impact of the intervention on residents’ anxiety, suicidal ideation, sleep problems and quality of life, b) examine the longer term impact of the intervention on depressive symptoms at follow-up (3 months post treatment), c) explore the impact of the intervention on staff and family levels of knowledge of late-life depression, stress levels and efficacy in caring for residents, d) study the impact of ELATE on MHT’s competency in geropsychology and e) evaluate the cost-effectiveness of the intervention model for the RAC sector.

## Methods and analysis

**Fig. 1:**
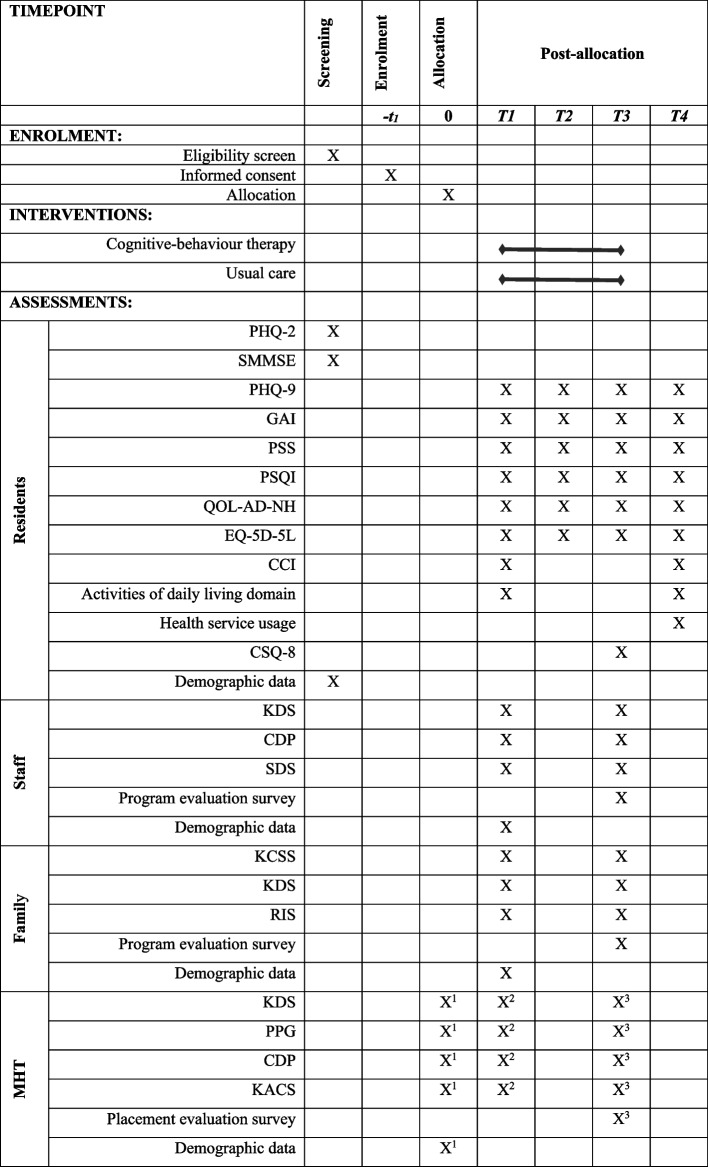
Schedule of enrolment, interventions and assessments. Note: S = Screening, T1 = baseline (prior to randomisation), T2 = mid-treatment (2.5 months post randomisation), T3 = post-treatment and primary endpoint (5 months post-randomisation), T4 = 3-month follow-up (8 months post randomisation), X^1^ = pre-training time point, X^2^ = post-training time point, X^3^ = post-placement time point. For other abbreviations, see section for abbreviations

This protocol is presented following the Standard Protocol Items: Recommendations for Interventional Trials (SPIRIT) guidelines [42] to ensure that all relevant information is included. A checklist of such information is provided (see SPIRIT checklist 2013, Additional file 1). The participant timeline recommended by SPIRIT [42] shows the schedule of enrolment, interventions and assessments (see Fig. 1).

### Study design

The study is a single blind, two-arm, parallel design, cluster randomised controlled trial (RCT) comparing older adults living in RAC facilities, where those facilities are randomised to receive ELATE or usual care (UC). The UC condition was selected as the control given that few RCTs of psychological interventions have been conducted in RAC facilities. The cluster design was selected to prevent contamination as the intervention involves aged care residents as well as facility staff and residents’ families. Group allocation is blinded to research assistant assessors but not to the participants.

### Participants and recruitment procedure

Residents are recruited from RAC facilities in Melbourne, Victoria, Australia. RAC facilities within a 15 KM radius of Swinburne University (Hawthorn campus) are invited to submit an expression of interest to participate in the study. Facilities within this radius with at least 5 residents who are estimated by facility staff to meet study eligibility criteria (see below) are eligible to join the study.

Facility staff – usually a facility manager, clinical care coordinator, or lifestyle coordinator of participating RAC facilities - nominate residents without significant cognitive impairment who are suspected to have depressive symptoms. Nominations are determined by the staff member’s subjective judgement, a review of residents’ records over the past 6 months and/or administration of Patient Health Quetionnaire-2 [43]. Once nominated, residents are then screened for study inclusion and exclusion criteria by a research assistant (RA).

Inclusion criteria for residents are: a) aged 65 years or above, b) significant depressive symptoms as indicated by Patient Health Questionnaire-2 [43] score of 3 or greater, and c) normal cognition or mild cognitive impairment as indicated by the Standardised Mini-mental state examination [44] score of 21 or greater. Exclusion criteria are a) poor English language proficiency, as assessed by facility or research staff and b) inability to complete study questionnaires or procedures due to health conditions, as assessed by facility or research staff. Residents are not excluded if they have a diagnosis of dementia.

Consent to participate in the study is obtained from the resident in writing or verbally. Consent is obtained from residents’ legal guardian or designated power of attorney if preferred by the resident or if the resident is believed by facility staff to lack capacity to provide informed consent.

**Fig. 2:**
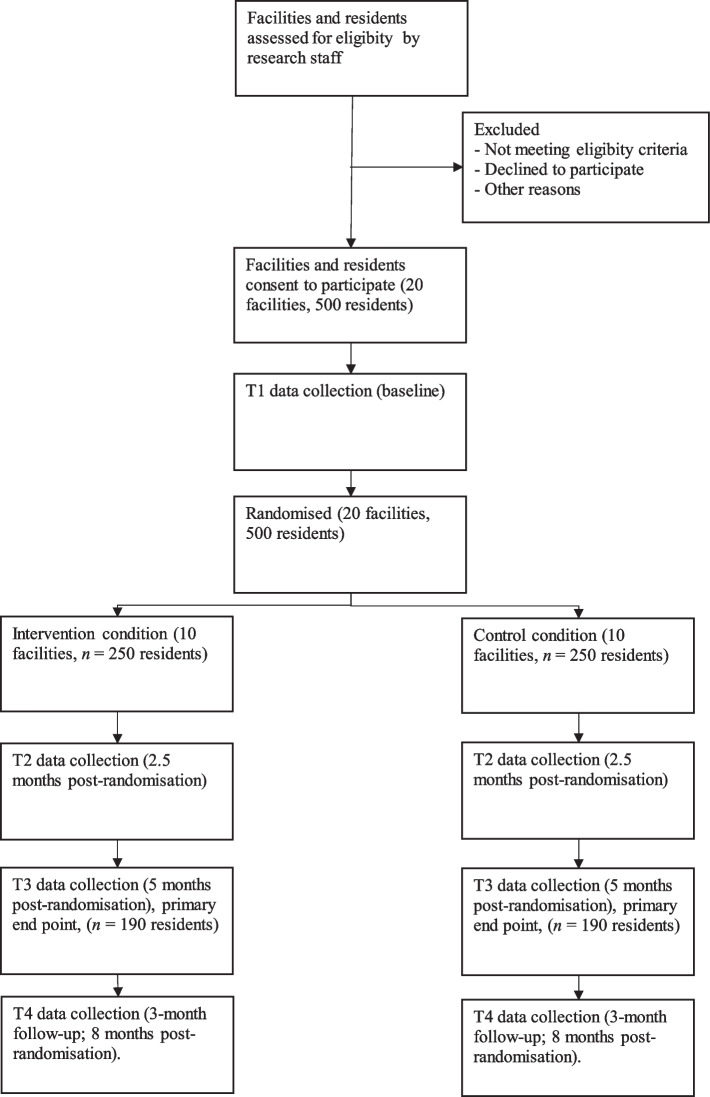
Consort flow diagram

We aim to recruit 500 residents from 20 RAC facilities in Melbourne. Estimations of sample size were based on expectations of an average of 25 participants in each facility, adjusted to allow for a within-site intra-class correlation of 0.05 [45] and an attrition rate over 5 months of 24%. The final sample of* n* = 380 participants (190 participants per condition) at the primary end-point (T3; 5 months post-randomisation), will provide 80% power to detect a mean improvement in depressive symptom sores of 3.0 (*SD* = 7.58) (as observed in our unpublished pilot data using the Cornell Scale for Depression in Dementia [46]) with a type 1 error rate of 5% and type 2 error rate of 20%. Figure 2 is a CONSORT (Consolidated Standards of Reporting Trials) diagram [47] of the primary participant flow anticipated through the trial.

This study also examines the impact of ELATE on RAC facility staff and residents’ family members. Residents who consent to the trial are asked to nominate a staff member as well as a family member (or a friend). An RA informs these nominees about the study and obtains written consent. Inclusion criteria are 1) staff member employed at the resident’ RAC facility or family member (or friend) of the resident, 2) nominated by the resident (or their proxy) as “an important source of support for the resident” (this term is deliberately open to allow for the nominator’s perspective), 3) aged 18 years or older and 4) sufficiently proficient in English, as assessed by facility staff or research team member.

In addition, the study examines outcomes for MHTs involved in the trial. MHTs are *postgraduate clinical psychology students* on clinical placement who deliver ELATE to residents in the study. MHTs are eligible to participate if they are a) aged 18 or over b) are enrolled in a masters or doctoral program in clinical psychology in Australia, c) have had experience in providing one-on-one counselling to clients, d) can commit to a 6-month clinical placement to deliver ELATE and e) pass the selection interview for the clinical placement. MHTs are supervised by clinicians experienced in mental health care of aged care residents.

### Randomisation

Following baseline assessments, RAC facilities are randomly allocated to the intervention or control condition. Randomisation through minimisation is allocated by the trial statistician (SQ) who is not involved in participant recruitment. Minimisation is based on the number of beds in a facility (Large > 74 beds v. Small ≤ 74 beds) and provider ownership (Privately owned, Non-privately owned). Following baseline assessment at the RAC facility, the statistician informs the project managers (JW, SD) of the allocation outcome for the site. Participants are informed of their allocation, but RAs conducting assessments are blinded to group allocation.

### Intervention

ELATE is an individually tailored intervention that is delivered over 5 months using a structured manual. RAC facilities randomised to the intervention condition are provided with ELATE in addition to usual care (described below). ELATE is designed to be suitable for residents with varying levels of cognitive and physical impairment, and to be delivered by MHTs to residents in collaboration with facility staff and family members. ELATE involves *weekly individual sessions with residents*, as well *activities involving facility staff and families*. RAC facilities allocated to the intervention condition receive both components. Intervention activities are completed face-to-face, but may be substituted with telehealth (video-conference or phone) when face-to-face contact is restricted due to COVID or illness related policies at the facility.

#### Component 1: weekly individual sessions with resident

The program comprises 16 weekly individual sessions with residents at the RAC facility. Sessions last 15–60 min, depending on the needs and attention span of the resident. The treatment approach is CBT that includes behavioural, cognitive and reminiscence strategies. CBT is a problem solving based treatment that aims to assist residents to examine their current beliefs and behaviours and reflect on the past with a view towards solving current problems, revising beliefs and modifying behaviours. The treatment helps individuals reach goals, develop coping skills and improve beliefs related to self, others and circumstances.

The treatment package for residents comprises 6 modules (Table 1). The first module (sessions 1–2) orients the resident to the treatment and clarifies treatment goals. The second module (sessions 3–5) involves prompting the resident to reminisce about important and positive experiences. The third module (sessions 6–9) involves activity scheduling, where residents are encouraged to engage in actives that provide them with feelings of pleasure and/or mastery. The fourth module (sessions 10–12) provides residents with anxiety management techniques such as relaxation strategies. The fifth module (sessions 13–15) helps residents use cognitive restructuring techniques to identify, evaluate and correct unhelpful automatic thoughts. The final module (session 16) involves a review and consolidation of strategies and development of a relapse prevention plan.

**Table 1:** Treatment package for residents

Module	Aim	Sessions
1. Introduction	This module orients the resident to therapy and establishes therapy goals through skilful assessment.	1, 2
2. Reminiscence	This module merges history taking with a reminiscence approach. It addresses previously utilised coping strategies and encourages residents to discuss positive aspects of their life history.	3, 4, 5
3. Behavioural activation	This module focuses on behavioural methods such as activity scheduling.	6, 7, 8, 9
4. Anxiety management	This module addresses anxiety by providing a rationale for how anxiety develops and is maintained, and by introducing relaxation training.	10, 11, 12
5. Cognitive restructuring	This module introduces structured cognitive therapy techniques in the form of identifying unhelpful beliefs and applying coping self-talk to commonly encountered frustrating situations in residential aged care facilities.	13, 14, 15
6. Relapse prevention	The sixth module addresses relapse prevention strategies in creating wellness plans.	16

MHTs are permitted to deviate from the treatment manual by changing the order of modules, varying the number of sessions in a module and eliminating a module, in order to tailor the treatment to match the resident’s treatment goals and capabilities. Such deviations are carefully documented and made in consultation with the clinical supervisor, assessment of the resident’s problems and discussions with family members and facility staff.

#### Component 2: activities involving facility staff and families

Facility staff and residents’ family (or friends) whom residents consider to be important sources of support, are offered three types of activities (Table 2). First, MHTs deliver a 1-h group workshop to residents’ family and a separate 1-h group workshop to facility staff members, usually within the first month of ELATE. These workshops are conducted face-to-face at the RAC facility or virtually (video conference). The workshop provides a) information about ELATE, b) an overview of depressive symptom and anxiety, c) an introduction to evidence based strategies for helping residents manage such symptoms and d) resources for supporting aged care residents. In addition, families receive the Beyond blue guide for carers [48] and facility staff receive a web link to Beyond Blue’s Professional Education to Aged Care (PEAC) [49].

**Table 2:** Involving family (or friends) and facility staff in ELATE

Systemic activity type	Aim	Frequency
Information and educational workshop	The aim of this 1-h educational workshop is to provide information about ELATE, depressive symptoms, and anxiety and to suggest strategies and resources for supporting residents.	Once, at the start of ELATE
Check in	The aim of this activity in to exchange information about a resident with their facility staff member and family member (or friend) for the purpose of a) learning about the perspective of facility staff and family about residents’ history, personality, strengths and difficulties, b) providing updates on the treatment plan and outcome, and c) facilitating staff and family to support the resident between treatment sessions.	Once a month
Joint sessions	The aim of this activity is to involve the staff and family (or friend) in joint sessions with the resident, when relevant. For example, it may be helpful to involve family or staff in sessions with the resident to brainstorm solutions to a problem.	When indicated

Second, the MHT consults individually with the resident’s family member (or friend) and facility staff once a month, in person, by phone or video conference to discuss care approaches that will meet residents’ needs and that can be feasibly implemented in the RAC facility. Each ‘check in session’ lasts 5–60 min. These sessions aim to a) assist the MHT consider family and staff perspectives on the resident; b) update family and staff on the treatment plan and outcome; and c) facilitate family and staff to support the resident between treatment sessions. MHTs seek residents’ consent prior to these sessions. These sessions also provide an opportunity for the MHT to acknowledge stressors involved in caring for residents, and to provide resources and referral options to support family and facility staff.

Third, MHTs invite family members and facility staff to attend some sessions with the resident. We anticipate that approximately 25% of sessions will be joined by family members and/or staff. Joint sessions are collaboratively pre-arranged with the resident and are held when considered beneficial for treatment. For example, families may be invited to attend sessions to assist residents in collating photographs for inclusion into an album highlighting the resident’s important experiences. Staff members may be asked to attend sessions to help organise activities for residents. Family and staff may also attend sessions to learn strategies that they can remind residents to use between sessions (e.g., relaxation techniques, cognitive restructuring).

### Treatment integrity

To maintain and monitor adherence to the intervention protocol, MHTs are provided with individual (one-on-one) clinical supervision and group supervision, on alternating weeks. Supervision is conducted face-to-face, by phone or by video-conference. Prior to commencing treatment with residents, MHTs complete 3 weeks of online training and attend an orientation meeting with the project coordinators (face to face, or by video-conference). Treatment sessions with residents are audio recorded with permission. Clinical supervisors rate a minimum of 5% of these recordings for treatment adherence and therapist competence. Clinical supervisors and MHTs also independently self-rate the MHT’s adherence and competence in delivering each treatment module, using a purpose-built treatment integrity checklist. In addition, residents’ attendance rates, frequency of sessions and length of sessions are recorded, as are the attendance of family and facility staff at monthly check-in meetings, joint sessions and workshops.

### Control condition

Participants in the control condition do not receive ELATE, but continue to receive standard care practices, including assistance with activities of daily living, medical care, scheduled group activities and prescribed psychotropic medications. In addition, the facility manager is provided an online booklet on psychological interventions for promoting emotional wellbeing in older adults [50] for distribution to facility staff.

### Measures

The primary outcome is the residents’ level of depressive symptoms, as measured by the Patient Health Questionnaire-9 (PHQ-9) [43], a reliable and valid measure for such symptoms in older adults [51] including those living in RAC facilities [52]. It comprises 9 questions that map the 9 criteria for a major depressive episode [53]. Items are rated on a 4-point Likert scale ranging from 0 (“Not at all) to 3 (“Nearly every day”). Item scores are summed to constitute a total score. The total score ranges from 0 to 27, with higher scores indicating increased depressive symptoms within the past 2 weeks The PHQ-9 is sensitive to changes in depressive symptoms over time [54], and has been commonly used to measure such changes in trials with older adults.

Secondary outcomes for residents are anxiety, suicide ideation, sleep problems and quality of life. Anxiety is measured by the Geriatric Anxiety Inventory (GAI) [55], comprising 20 “Agree/Disagree” items designed to assess anxiety symptoms in older adults. Suicide ideation is measured by the Paykel Suicide Scale (PSS) [56], which comprises 5 Yes/No items that measure suicide desire, intentions and attempts over the past week. Sleep problem**s** are measured by the Pittsburgh Sleep Quality Index (PSQI) [57], a 19-item measure of sleep quality and disturbances over a 1-month time interval. Quality of life is assessed in two ways - by the EQ-5D-5L instrument [58] which is a measure of health related quality of life, developed for health economic evaluation, and by the Quality of Life in Alzheimer’s Disease Scale-Nursing Home Version (QOL-AD-NH) [59], a 15 item self-rated measure specifically designed for use within RAC settings.

The potential benefits of ELATE for facility staff, families and MHTs are also assessed. Knowledge of late life-depression by facility staff, families and MHTs is assessed using the Knowledge of Late-Life Depression Scale-Revised [60], comprising 10-items measuring knowledge about symptoms, myths and facts of late life depression. Stress levels of families and facility staff are measured with the 10-item self-report Kingston Caregiver Stress Scale [61] and 27-item self-rated Strain in Dementia Care Scale [62], respectively. Self-efficacy for caring for residents by families is measured with the 10-item self-rated Relational, Instrumental and Self-care Eldercare Self-efficacy Scale (RIS) [63]. Confidence of staff and MHTs in working with aged care residents with depressive symptoms is measured using the 14-item self-rated Confidence in Working with Depressed Older People Scale [64]. Geropsychology competencies and attitudes in MHTs are measured using the 50-item self-rated version of the Pikes Peak Geropsychology Knowledge and Skill Assessment Tool (PPG) [65] and a purpose-built 14-item self-rated measure: Knowledge, Attitudes and Confidence in Working with Older Adults Scale (KACS).

In addition to outcome measures, resident cognition is screened using the Standardised Mini Mental State Examination [44]. Resident demographics - gender, date of birth, languages spoken, and highest level of education - are also recorded at screening. Residents’ medical history and functional ability are rated by facility staff at baseline and T4, by completing the 15-item Charlson Comorbidity Index (CCI) [66] and Activities of Daily Living Domain of the Australian Aged Care Funding Instrument (11 items, [67]). At T4, residents’ health service usage over the study period are obtained from facility staff. These usage data are: hospital admission (number, reasons, and duration), presentations at emergency departments (number, reasons) and mental health treatments (types, and number of visits). Additional information about medical visits, hospitalisations and medications between T1 and T4 are accessed from three government administrative datasets: Medicare Benefits Schedule (MBS) [68], Pharmaceutical Benefits Schedule (PBS) [69] and the Centre for Victorian Data Linkage (CVDL) records [70].

Treatment acceptability by residents is assessed at T3 using the Client Satisfaction Questionnaire-8 (CSQ-8) [71], a validated 8-item self-report measure of treatment satisfaction. Each item is answered using a 4–point Likert scale to indicate the respondent’s satisfaction with various aspects of the treatment (e.g., quality, type, amount). In addition, residents are asked 5 open-ended questions about their perceived benefits and negative experiences in participating in the intervention as well as their recommendations for improving the program. At T3, participating family and aged care staff are also invited to complete a 13-item Program Evaluation Survey to indicate their level of involvement in, and satisfaction with, intervention activities. Following their clinical placement, MHTs indicate their satisfaction in relation to the training workshops, supervision and placement through a 34-item Placement Evaluation Survey.

### Data collection and management

Figure 1 shows the time points when each measurement is conducted for the different participant cohorts. Residents participating in the trial are assessed at four time points: baseline prior to randomisation (T1), mid-treatment (T2; 2.5 months post randomisation), post-treatment (T3; 5 months post-randomisation) and follow-up (T4; 8 months post randomisation). Facility staff and residents’ families are assessed at T1 and T3. MHTs are assessed at three time points: pre-training (prior to training), post-training (after training, but prior to resident contact) and post-placement (at completion of their clinical placement).

RAs are trained to administer measures to residents through semi-structured interviews. Assessment interviews with residents are digitally recorded. A second RA listens to a minimum of 10% these recordings and rates measures independently. The two sets of ratings are compared to assess inter-rater reliability. Further, following the interviews, RAs self-rate the extent to which they remain blinded with respect to the participants’ group allocation. Hard copy data are stored in a coded (re-identifiable) form in a locked filing cabinet at Swinburne University. Electronic data are coded (re-identifiable) and stored on password protected computer systems at Swinburne University. Trial data are only accessible by the research team and are retained for at least 7 years after the last published outcome. Facility staff and family participating in the study provide data by completing self-report measures online, in hardcopy form or by interview (over the phone or by video-conference). MHTs provide data by completing online self-report measures.

Participants can withdraw from the study at any time. Data collected from them are retained confidentially by the project team and included in the analyses, unless otherwise instructed by participants. A data monitoring committee was not considered necessary as the trial is not testing a drug or device and any safety concerns associated with the trial are regularly reported to, and reviewed by, the university ethics committee.

### Data analysis

Statistical analyses will be conducted on an intention-to-treat basis. Missing data will be imputed using multiple imputations, redrawing 50 samples. The primary outcome is change in depressive symptoms between conditions between T1 and T3, as measured by the PHQ-9. The primary analysis is a regression, clustered over site, to account for correlated readings. The independent variables are condition and depression at baseline. The secondary variables, anxiety, suicide ideation, sleep problems and quality of life will be treated similarly. Multilevel random effects mixed modelling will be conducted as a sensitivity analysis for each outcome, measured at all four time points. Individual and site will be entered as random effects. Time will be coded in months from baseline and parametrised as appropriate to improve model fit. The effect will be assessed using the interaction term between time and condition. Standard checks of homoscedasticity and normality of residuals at each level will be conducted as well as independence of residuals between levels to verify model validity. T-tests or chi-squared tests will be used as appropriate to compare demographic imbalances between conditions. Analyses will be conducted using Stata [72].

#### Economic evaluation

An economic evaluation will be conducted to assess the cost-effectiveness of ELATE. The economic evaluation will use a cost-utility analysis framework, where the incremental costs associated with the intervention will be combined with the incremental benefits expressed as quality adjusted life years (QALYs) generated from responses to the EQ-5D-5L. These responses will be converted into utility scores, anchored on a scale between 0 (indicating a health state equivalent to being dead) and 1 (indicating full health) using available preference-weights generated from a general population sample [73]. These utility scores will be converted into QALYs gains for each individual participant over the follow-up period by combining data regarding the health states of the participants with information on the time spent in those health states, using area under the curve methods [74].

The resources associated with administration of ELATE will be measured and valued using appropriate unit costs. Health service utilisation data for duration of the trial such as in-patient hospital admissions, primary health care services, prescribed pharmaceuticals, and emergency department presentations will be obtained from three routine administrative government datasets (PBS, MBS, and CVDL) supplemented with RAC facility records. Unit costs will be derived from publicly available data sources and as recommended by guidelines for conducting economic evaluations [68–70, 75, 76]. Mean differences in the costs and outcomes for the two groups will be presented [77].

As the primary economic analysis, we will present the incremental cost-effectiveness ratio which will be the measure of additional cost for each QALY gained via the intervention. As a secondary analysis we will use clinical outcomes for the trial as the measure of benefit within a cost-effectiveness analysis framework. The uncertainty regarding the cost-effectiveness of the intervention will be presented using cost-effectiveness acceptability curves. An assessment of the sensitivity of the results obtained, to variation in measured resource use, effectiveness and/or unit costs, will be undertaken using appropriate one-way and multi-way sensitivity analysis [78, 79].

## Discussion

This study protocol presents the design of a cluster RCT that aims to examine the effectiveness of ELATE - a CBT program for reducing depressive symptoms, specifically designed for RAC populations. Most psychological treatments for late-life depressive symptoms have been evaluated with community-based samples and hence may not be accessible and feasible for implementation in RAC settings. Generalising findings from community to RAC settings is problematic as residents are older, more cognitively impaired, more physically limited and hence more dependent on others for day-to-day assistance, compared to community dwelling older adults [24, 25]. Hence, residents do not access publicly available psychological treatments for depressive symptoms [17], resulting in an over-reliance on pharmacological approaches in such settings [11, 12].

ELATE is designed to be feasible for RAC populations. The treatment program is systemic, in that therapists collaborate with facility staff and residents’ families to deliver psychological strategies to residents. The program is integrative, as it draws on behavioural, cognitive and reminiscence strategies to accommodate residents with various levels of cognitive and physical impairment. The program is delivered by trainee therapists under supervision of experienced practitioners, thus providing a possible cost-effective model of mental health care in RAC facilities. The intervention model builds on effective strategies for assisting older adults with depression, whilst also addressing the need to support and train families, facility staff and trainee therapists in recognising and responding to the mental health care needs of residents.

While psychological interventions have been found to be beneficial for older adults with depression who live in the community, there is a dearth of studies on the effectiveness of such treatments in residential settings. In addition, while some studies have shown the benefits of such interventions to RAC residents when delivered by qualified clinicians, more research is needed regarding whether such effects can be achieved when such interventions are delivered by trainee therapists and the extent to which this model is cost-effective for the RAC sector. If found beneficial and cost-effective, ELATE can be widely disseminated across this sector.

### Dissemination

There will be no limitations to the dissemination of results. It is anticipated that the results of the study will be published and presented in various forums, including peer reviewed journal publications, conference presentations, aged care industry communications and media releases. In all publications and presentations, participants will not be identified.

### Supplementary Information

**Additional file 1.** SPIRIT 2013 Checklist: Recommended items to address in a clinical trial protocol and related documents*.

**Additional file 2: Supplementary Table 1.** CONSORT 2010 checklist of information to include when reporting a cluster randomised trial. **Supplementary Table 2.** Extension of CONSORT for abstracts to reports of cluster randomised trials.

## Data Availability

Resultant data sets may be made available upon reasonable request of the corresponding author once the study is concluded.

## Abbreviations

CBT: Cognitive behaviour therapy
CCI: Charlson Comorbidity Index
CDP: Confidence in Working with Depressed Older People Scale
CONSORT: Consolidated Standards of Reporting Trials
CSQ-8: Client Satisfaction Questionnaire-8)
CVDL: Centre for Victorian Data Linkage
ELATE: Elders at Ease
GAI: Geriatric Anxiety Inventory
KACS: Knowledge, Attitudes and Confidence in Working with Older Adults Scale
KDS: Knowledge of Depression Scale-Revised
KCSS: Kingston Caregiver Stress Scale
MBS: Medicare Benefits Schedule
MHTs: Mental health trainees
PBS: Pharmaceutical Benefits Schedule
PHQ-2: Patient Health Questionnaire - 2 item version
PHQ-9: Patient Health Questionnaire - 9 item version
PPG: Pikes Peak Geropsychology Knowledge and Skill Assessment Tool
PSS: Paykel Suicide Scale
QALY: Quality adjusted life year
PSQ: Pittsburgh Sleep Quality Index)
QOL-AD-NH: Quality of Life in Alzheimer’s Disease Scale-Nursing Home Version
RA: Research Assistant
RAC: Residential aged care
UC: Usual care
RCT: Randomised controlled trial
RIS: Relational, Instrumental and Self-care Eldercare Self-efficacy Scale
S: Screening
SDS: Strain in Dementia Care Scale
SMMSE: Standardised Mini-mental state examination
SUHREC: Swinburne University Human Research Ethics Committee
T1: Baseline assessment (prior to randomisation)
T2: Mid-treatment assessment (2.5 months post randomisation)
T3: Post-treatment assessment and primary endpoint (5 months post-randomisation)
T4: 3-Month follow-up (8 months post randomisation)
X^1^: Pre-training time point
X^2^: Post-training time point
X^3^: Post-placement time point
